# The Book World of Medicine and Science

**Published:** 1911-09-30

**Authors:** 


### The Book World of Medicine and Science.

Food and Feeding in Health and Disease. By Chalmers Watson, M.D., F.R.C.P.E. (Edinburgh: Oliver and Boyd. Price 10s. 6d. net.)

The practitioner should have on his shelves at least one standard volume dealing with the important subject of dietetics. Without offering an opinion on the merits of the various treatises which he may buy to serve that end, we may safely say that he will be well advised to expend half a guinea in purchasing this work by so well-known an authority as Dr. Chalmers Watson, whose researches on the values of different articles of diet deserve wider recognition than they have yet obtained. We have read the volume with interest, and have found it both scientific and practical. Its usefulness to the practitioner it would be difficult to over-estimate, for not only does it give detailed accounts of the various articles of diet, but it abounds in practical hints, recipes, and suggestions, while the appendix, which gives an account of the researches carried on in the physiological laboratory of Edinburgh University on the influence of a wholly meat diet, is not the least valuable part of the book. Such points as idiosyncrasy, rectal feeding, milk contamination, diets in various diseases, beverages, vegetarianism, and animal foods are fully discussed, and the information given, while it is often original, is always instructive and of practical utility. The book should be a useful adjunct to the physician's select library, and laymen who are unacquainted with the physiological subtleties of dietetics will find much in it that they can appreciate and understand. Summed up, it is one of the best books of the year. Written in an attractive fashion, that nowhere proves boring but is throughout interesting, it is excellent reading and may be whole-heartedly commended to the attention of practitioners and students.

Laboratory Studies in Tropical Medicine. By C. W. Daniels, M.B., M.R.C.P., and H. B. Newham, M.R.C.S., L.R.C.P., D.P.H. (London: John Bale, Sons, and Danielsson, Ltd. 16s. net.)

We favourably reviewed the second edition of this popular text-book, which is one of the best laboratory manuals on tropical medicine. The third reprint has been thoroughly revised, and is fully up to date in every respect, containing much additional matter, so that the book is one of the most complete compendiums which the student of tropical medicine can obtain. Essentially practical, it abounds in hints, and is therefore suitable for the post-graduate worker who endeavours to master the subjects for the D.T.M. syllabus on "a lone hand." Details of various methods of staining are clearly and precisely explained; the somewhat uncertain and protean classification of the parasites is given with as much accuracy as is possible in view of the known instability of existing classifications and uncertainty with regard to types; clinical methods, such as the preparation of charts and blood films, are fully described, and the book abounds with really helpful and accurately drawn illustrations. We know of no better manual in which the medical man who is proceeding abroad or who intends to take a course in tropical medicine can invest.

The Reduction of Domestic Mosquitos. By Edward Halford Ross, M.R.C.S. (London: John Murray, 1911. Price 5s.)

That such a book as this should be called for at the present stage of our knowledge of the mosquito is nothing less than an indictment of the slow progress made by sanitary science in some communities. It is quite ten years ago since the importance of destroying mosquitos was pointed out by Professor Ronald Ross and the substantial benefits to be gained were demonstrated to municipal authorities. Yet it has been found advisable and no doubt necessary, to return to the attack and to bring again to public notice the malevolent properties of all kinds of mosquitos, not only to malaria disseminating ones, and to show how and wherefore these pests should be destroyed in the interests of public health. Dr. E. H. Ross has endeavoured to drive home the practical aspects of organising a mosquito campaign, and in this book of his we find he has, even at the cost of some repetition, entered into all those details to which attention is so absolutely necessary for such a campaign to succeed. If at times the author indulges in language bordering on the picturesque or dwells unnecessarily on the obvious, it must be admitted that he is terribly in earnest and that his cause is worthy of his enthusiasm. He does not minimise the difficulties to be encountered, but deals with them in a thoroughly practical spirit and with full acquaintance of the value of tact in dealing with the failings of human nature. Perhaps the book would have been more potent for good had it been shorter, but nevertheless it will, we trust, be productive of much benefit in educating public opinion in mosquito-ridden localities and in inciting authorities to tackle this problem—a problem, too, which it will pay financially to tackle thoroughly. With the examples before them of what has actually been accomplished in Port Said and other places, and with the practical hints obtained from former experience, it is sincerely to be hoped that substantial progress will be made in overcoming the inertia of public bodies and the ignorance and prejudice of the populace. Many excellent plates increase the value of Dr. Ross' book, some of which form instructive object-lessons.

Joint Tuberculosis. By Leonard W. Ely, M.D. (Bristol: John Wright and Sons, Ltd. 1911. Price 12s. 6d. net.)

This is a most interesting and readable book on a subject which has for long clamoured for adequate text-book treatment. We cannot regard Dr. Ely's work as an exhaustive text-book, comparable, for example, with the elaborate monographs in Deutsche Chirurgie: his space is too limited to make it that. But as a supplementary manual to the ordinary work on surgery it is undoubtedly of the greatest use to practitioners and students. The general plan of the book is very simple. Starting with an exposition of what may be called the fundamentals of joint tuberculosis, the author proceeds, in the first section, to deal with the general elements of treatment, pathology, and ætiology. Here his views are on the whole sound, though they cannot claim to be very original. Personally we feel that he has not quite done justice to the Bier-Klapp method of treating closed cases of joint tubercle, and that he is inclined to lay too much stress on the importance of fixation and immobilisation—the latter of which is admittedly impossible in certain cases. In the second part, special lesions—regional tubercle—are dealt with, each type being discussed under the headings of incidence, symptoms, and treatment. Here the author takes too much for granted. How many English readers will know what a Lorenz stirrup is, for example? It is one of the most generally useful lower-leg splints we have, but it is practically unknown in English practice. More detailed accounts of symptomatology might have been given in some cases, especially in the rarer examples where the difficulty of differentiation is a very real one. For instance, in the short chapter dealing with sacroiliac disease the author omits the useful Trendelenberg's sign which, as Spitzy has shown, is often present in these cases. The book, taken as a whole, is practical, and certainly demands the consideration of the general practitioner. In a second edition we hope the author will point out the usefulness of the celluloid pot in cases of joint tubercle—an aid which is much less widely known than it deserves to be.

Surgery. Part I. Catechism Series. Second edition. Revised and enlarged. With illustrations. (Edinburgh: E. and S. Livingstone, 1911. Price 1s. net.)

It can only be presumed that the method of presenting knowledge in the form of catechisms still retains a considerable vogue among medical students, for this series of booklets is continually being revised. The sample under consideration is certainly good as far as it goes, but we must be allowed to marvel how such a book could be of much use to a student "on the eve of examination"; we could more easily conceive of its utility to him during his first month of hospital work. This edition has been modernised here and there; we find $CO_2$ snow mentioned under Naevi, but pages are devoted to almost mediæval operations for aneurysm.

